# Beyond Inflammation: Why Understanding the Brain Matters in Inflammatory Arthritis

**DOI:** 10.1002/acr.25694

**Published:** 2025-11-14

**Authors:** Eoin M. Kelleher, Rosario Meouchi, Anushka Irani

**Affiliations:** ^1^ University of Oxford Oxford UK; ^2^ Massachusetts General Hospital Boston; ^3^ Mayo Clinic Florida Jacksonville

## Abstract

Persistent pain remains a major challenge in inflammatory arthritis, even when joint inflammation is well controlled. Pain and associated symptoms such as fatigue cannot be explained by peripheral inflammation alone but reflect altered central pain processing. These changes may arise through “top‐down” mechanisms, reflecting pre‐existing dysfunction in pain perception, or “bottom‐up” pathways, driven by peripheral inflammation acting on the brain. Neuroimaging has transformed understanding of these processes by providing in vivo markers of how brain function and structure are related to pain. Functional magnetic resonance imaging (MRI) demonstrates that both task‐evoked and resting‐state activity are altered in inflammatory arthritis. Connectivity changes involving the thalamus, insula, medial prefrontal cortex, and default mode and salience networks correlate with pain, fatigue, and affective symptoms. Notably, tumor necrosis factor α (TNF‐α) inhibitors rapidly normalize pain‐related activation, preceding improvements in joint swelling, strongly supporting a bottom‐up role for peripheral inflammation. Recent randomized controlled trial data show that baseline central nervous system pain activation predicts analgesic response to TNF‐α blockade, positioning neuroimaging as a potential tool for treatment stratification. Complementary modalities provide further insights. Proton electron tomography studies suggest altered pain responses, and novel tracers may clarify contributions of neuroinflammation. Magnetic resonance spectroscopy reveals neurochemical correlates such as increased choline and myo‐inositol linked to fatigue, although group‐level evidence for overt neuroinflammation remains limited. Structural MRI highlights gray matter changes in regions mediating sensory, cognitive, and affective processing. Together, this supports a dual top‐down and bottom‐up model of persistent pain in inflammatory arthritis, with important implications for mechanism‐based therapies targeting both immune and brain pathways.

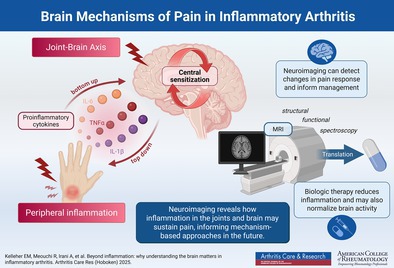

## Graphical abstract

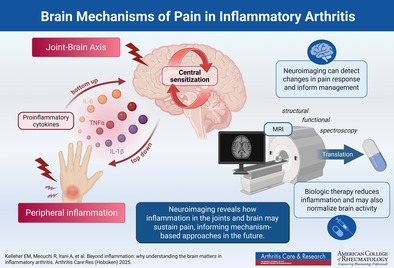

## Introduction

Pain is the cardinal symptom of inflammatory arthritis and remains a top priority for patients living with conditions such as rheumatoid arthritis (RA), psoriatic arthritis (PsA), and axial spondyloarthritis (axSpA).^1^, ^2^ Despite significant advances in pharmacologic therapies targeting immune pathways, many patients continue to experience persistent pain even when inflammatory joint disease is apparently well controlled.^3^

Although inflammation contributes to pain intensity, improved control of synovitis has not consistently translated into meaningful reductions in pain or functional limitation over time.^4^, ^5^ This clinical disconnect highlights a critical unmet need and calls into question long‐standing assumptions that ongoing pain necessarily directly reflects active joint inflammation.

The emergence of the “difficult‐to‐treat” RA concept underscores this challenge,^6^, ^7^, ^8^ estimated to affect 14.4% of patients with RA and up to 22.3% of those treated with biologics.^9^ Patients may present with persistent symptoms suggestive of disease activity despite adequate immunomodulatory therapy with multiple conventional and biologic disease‐modifying antirheumatic drugs (DMARDs). Importantly, recent efforts to subcategorize these patients into **inflammatory‐refractory RA** and **noninflammatory RA** ^6^ recognize the possibility of distinct underlying pain mechanisms.^10^, ^11^

This mechanistic reframing is mirrored in the updated chronic pain taxonomy introduced by the International Association for the Study of Pain (IASP) and adopted into the *International Classification of Diseases, 11th Revision*.^12^ Chronic pain is now classified as either chronic primary (eg, fibromyalgia) or chronic secondary (eg, pain because of inflammatory arthritis), with the latter designation capturing cases in which pain persists independently of active inflammation. This classification has the potential to inform more tailored treatment strategies for patients with inflammatory arthritis and coexisting chronic pain.

Importantly, multiple pain mechanisms can coexist in individuals with inflammatory arthritis. The IASP also proposes three mechanistic descriptors: nociceptive, neuropathic, and nociplastic pain.^13^, ^14^ Nociceptive pain arises from tissue damage and is typical in active synovitis. Neuropathic pain, caused by somatosensory system lesions, is relatively uncommon in these conditions, although features of neuropathic pain may be present.^15^ Nociplastic pain reflects altered central pain processing without clear evidence of ongoing tissue damage or nerve injury and may underlie persistent pain in patients with well‐controlled inflammation. This category overlaps conceptually with previously used terms, such as “central sensitization” or “centralized pain,” with fibromyalgia serving as a prototypical example.^11^, ^13^ Recent large‐scale neuroimaging data further support the biologic validity of nociplastic pain. In a population‐based study of >40,000 participants in UK Biobank, we demonstrated that higher Fibromyalgia Index scores, reflecting greater nociplastic pain burden, were associated with altered structural connectivity among the periaqueductal gray, amygdala, and hypothalamus, key nodes of the descending pain modulatory system (DPMS).^16^ These findings provide population‐level evidence for central pain amplification mechanisms.

There is accumulating evidence to suggest that persistent pain in inflammatory arthritis may have a nociplastic, in addition to nociceptive, component. Proinflammatory cytokines may influence neural activity at both peripheral and central levels.^17^, ^18^ Neuroimaging offers a promising avenue to explore these phenomena in vivo, allowing noninvasive assessment of brain mechanisms underlying pain and other centrally mediated symptoms.^19^, ^20^, ^21^

This review aims to summarize key neuroimaging findings related to inflammatory arthritis pain, examine how current treatment strategies influence central pain processing, and propose a more integrated, mechanism‐based clinical model to guide care. By recognizing the brain as central to inflammatory arthritis pathophysiology, we advocate a shift from inflammation‐focused “treat‐to‐target” strategies toward a broader goal of “treat‐to‐recovery,” emphasizing restoration of function and quality of life.

## Imaging the pain experience: a window into the brain

Advances in neuroimaging have provided powerful tools to investigate the neural mechanisms underlying pain in inflammatory arthritis. These tools offer complementary perspectives on how the brain processes pain and related symptoms such as fatigue, mood disturbance, and cognitive dysfunction, creating a multidimensional picture of the pain experience.

### Functional magnetic resonance imaging

Blood oxygenation level–dependent (BOLD) functional magnetic resonance imaging (fMRI) is the most widely used method to study pain‐related brain activity. Task‐based fMRI can reveal how the brain responds to experimentally evoked pain, often showing increased activation in key pain‐processing regions such as the insula, anterior cingulate cortex (ACC), and thalamus. Resting‐state fMRI (rs‐fMRI), by contrast, measures spontaneous connectivity among brain regions, allowing assessment of how large‐scale networks such as the default mode network (DMN), salience network, and frontoparietal network interact in chronic pain states.

### Positron emission tomography

Positron emission tomography (PET) provides complementary information on brain function and, with specific radiotracers, can assess both regional blood flow and markers of neuroinflammation. In chronic pain conditions, PET has highlighted altered nociceptive processing and, with newer tracers, evidence of glial activation, suggesting a contribution of neuroimmune mechanisms to persistent pain.^22^

### Magnetic resonance spectroscopy

Magnetic resonance spectroscopy (MRS) enables in vivo assessment of brain neurochemistry by quantifying metabolites such as choline, myo‐inositol, glutamate, and γ‐aminobutyric acid. These measures can provide insights into cellular processes including glial activity and neurotransmission, which are thought to play a role in sustaining chronic pain.

### Structural MRI

High‐resolution structural MRI allows volumetric analysis of gray and white matter, offering insight into long‐term brain changes. Alterations in regions involved in pain appraisal, affective processing, and sensorimotor integration have been reported across chronic pain populations.

Together, these modalities provide a multidimensional view of the pain experience in inflammatory arthritis, from real‐time network activity and connectivity to underlying neurochemical and structural changes. By integrating these approaches, we can begin to unravel the complex interactions among inflammation, nociception, and central pain modulation.

## Pain pathways rewired: neuroimaging insights into central mechanisms in inflammatory arthritis

Neuroimaging studies provide compelling evidence that persistent pain in inflammatory arthritis is not just caused by peripheral inflammatory mechanisms but also involves central nervous system (CNS) pathways in its maintenance. These findings are particularly relevant for the subset of patients whose pain persists despite optimal control of joint inflammation.

### Evidence for nociplastic pain and overlap with fibromyalgia

**Figure 1:**
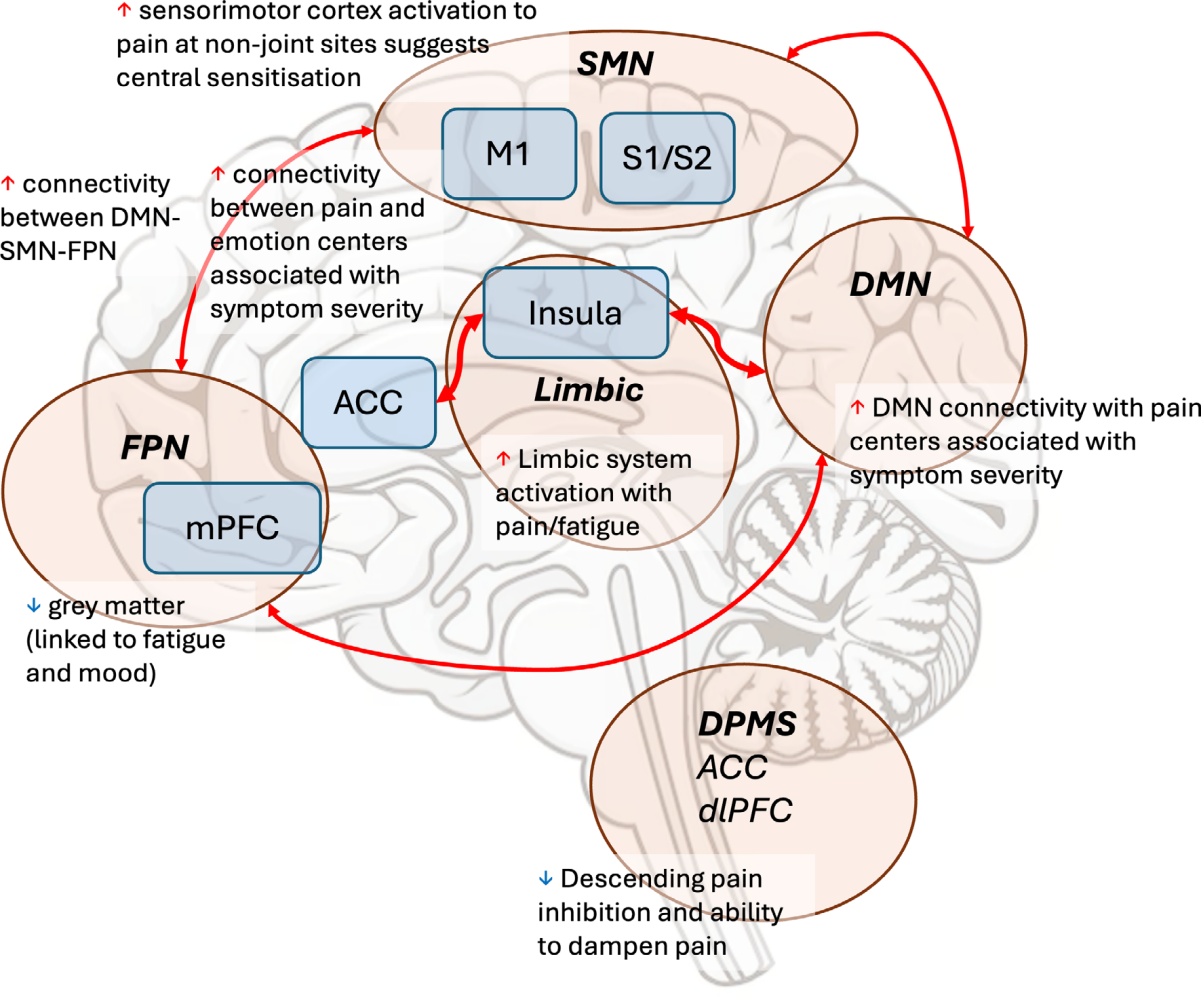
Brain mechanisms of nociplastic pain in inflammatory arthritis. Neuroimaging studies show that patients with inflammatory arthritis may experience persistent pain even when inflammation is controlled because of changes in how the brain processes pain. Brain networks that normally regulate sensation (SMN), thinking (FPN), and self‐awareness (DMN) become more strongly interconnected, contributing to fatigue and pain. Connections between pain and emotion centers (insula and ACC) are linked to symptom severity, whereas the limbic system shows greater activation with pain and fatigue. Exaggerated pain responses extend beyond inflamed joints, suggesting central sensitization. At the same time, brain regions that normally dampen pain (DPMS) show reduced activity. Shrinkage of gray matter in the mPFC further relates to fatigue and mood changes. These findings highlight brain contributions to nociplastic pain in inflammatory arthritis. ACC, anterior cingulate cortex; dlPFC, dorsolateral prefrontal cortex; DMN, default mode network; DPMS, descending pain modulatory system; FPN, frontoparietal network; mPFC, medial prefrontal cortex; SMN, sensorimotor network.

fMRI studies indicate that RA can represent a “mixed pain state,” in which nociplastic pain mechanisms in the CNS coexist alongside nociceptive drivers of pain from the joints (Figure 1). Basu and colleagues demonstrated that patients with RA with higher “fibromyalgianess” scores, even without meeting the diagnostic criteria for fibromyalgia, exhibited abnormal functional connectivity between the DMN and the insula, patterns previously identified as characteristic of fibromyalgia.^23^ Notably, these findings were associated not only with widespread pain but also with fatigue and cognitive dysfunction, underscoring the multisystem symptom burden often observed in these patients. These findings align with quantitative sensory testing data showing lower pain pressure thresholds across both affected and unaffected body regions in RA, consistent with central sensitization.^24^, ^25^ In a recent study in PsA, Sunzini and colleagues reported similar results, finding that fibromyalgianess scores correlated positively with resting‐state functional connectivity found between the right anterior insula and the DMN in rs‐fMRI.^26^ However, in contrast, studies by Flodin and colleagues and Sandström and colleagues found that CNS activation to evoked pain at nonjoint sites did not differ between those with RA and healthy controls.^27^, ^28^ This suggests that although pain sensitivity is increased in joint sites, there is not necessarily a generalized increase in peripheral pain sensitivity. However, it should be noted that these studies recruited patients with RA without comorbid fibromyalgia symptoms and thus may have excluded those patients with RA with more severe nociplastic features.

### Suggestion of neuropathic pain characteristics

Wu and colleagues investigated the presence of a neuropathic pain component in patients with ankylosing spondylitis (AS).^29^ Using structural MRI and the painDETECT questionnaire (a screening tool for neuropathic pain that was validated in patients with low back pain^30^) they found an inverse correlation between left primary somatosensory cortex thickness and painDETECT scores. Conversely, thickness of the left primary motor cortex, right ACC, and right dorsolateral prefrontal cortex (dlPFC) exhibited a direct correlation with painDETECT scores. Although definite neuropathic pain cannot be identified using the painDETECT alone, as this requires the confirmation of a somatosensory lesion,^31^ the association between these neuropathic‐like pain characteristics and changes in centrally mediated pain processing has been demonstrated by us and others in a variety of musculoskeletal conditions.^32^, ^33^, ^34^, ^35^

### Altered resting‐state connectivity and pain‐processing pathways

Resting‐state connectivity analyses further support the presence of widespread CNS reorganization in inflammatory arthritis. Flodin and colleagues observed increased PFC‐insula connectivity at rest in those with RA compared with healthy controls, however this was not itself associated with symptom severity, which may be caused by the relative insensitivity of rs‐fMRI in small sample sizes.^27^ Schrepf and colleagues reported that higher levels of systemic inflammation in RA were associated with enhanced connectivity among the medial PFC (mPFC), inferior parietal lobule (IPL), and canonical brain networks, including the DMN and dorsal attention network, during an attention task.^36^ These changes correlated with greater fatigue, pain, and cognitive dysfunction, suggesting that inflammation may act as a driver of maladaptive network changes in the CNS.^36^ Graph theoretical analyses extended these findings, revealing a distributed network of 49 regions linked to peripheral inflammation, measured by erythrocyte sedimentation rate (ESR), further implicating the IPL and mPFC as important nodes in the translation of inflammatory signals into central pain‐processing abnormalities.

These observations complement longitudinal imaging work demonstrating that anti–tumor necrosis factor (TNF) therapy reduces pronociceptive brain connectivity, in some cases preceding improvements in joint swelling and clinical indices of disease activity.^37^, ^38^ This is supported by the recent PreCePRA randomized multicenter clinical trial by Hess and colleagues,^39^ which investigated whether baseline brain activation on task‐based fMRI could predict treatment response to certolizumab pegol. Patients with RA were stratified by the extent of brain activation on fMRI in response to joint compression and randomized to anti‐TNF therapy or placebo. At 12 weeks, more than half of patients with high baseline CNS activation achieved low disease activity on TNF inhibition compared with less than half in the low‐activation group and only one‐quarter of those receiving the placebo. Importantly, the improved outcomes in the high‐activation group were largely driven by patient‐reported measures such as pain and global disease activity, rather than objective markers of inflammation, which improved similarly across treatment groups. Functional imaging further demonstrated that TNF blockade selectively reduced pain‐related activation in the primary somatosensory cortex, motor cortex, and frontal pole in the high‐activation group. These findings suggest that a strong “disease twin” in the CNS, reflecting heightened central representation of joint pain, may identify patients who are more responsive to TNF inhibition, supporting the concept that bottom‐up inflammatory signaling amplifies central sensitization. Conversely, patients with low CNS activation may have pain sustained more by top‐down processes and respond less to purely anti‐inflammatory strategies. These findings imply that TNF‐α may influence pain through mechanisms partially independent of its effects on synovial inflammation, with one pathway driving joint swelling and another promoting peripheral sensitization and central amplification of pain. Collectively, the previous observational work and this recent trial provide proof of concept that neuroimaging biomarkers could stratify patients with inflammatory arthritis for mechanism‐based treatment approaches.

Together, these neuroimaging insights illustrate that persistent pain in inflammatory arthritis cannot be solely explained by peripheral inflammation. Instead, aberrant connectivity across salience network, DMN, and frontoparietal network, as well as structural changes in regions such as the mPFC and IPL, highlight the CNS's active role in shaping the pain experience. Recognizing these mechanisms is important for rheumatologists, as they provide a rationale for integrating interventions that target pain processing in the CNS alongside traditional immunosuppressive strategies.

## When inflammation talks to the brain: the interaction between inflammation and central pain pathways

**Figure 2:**
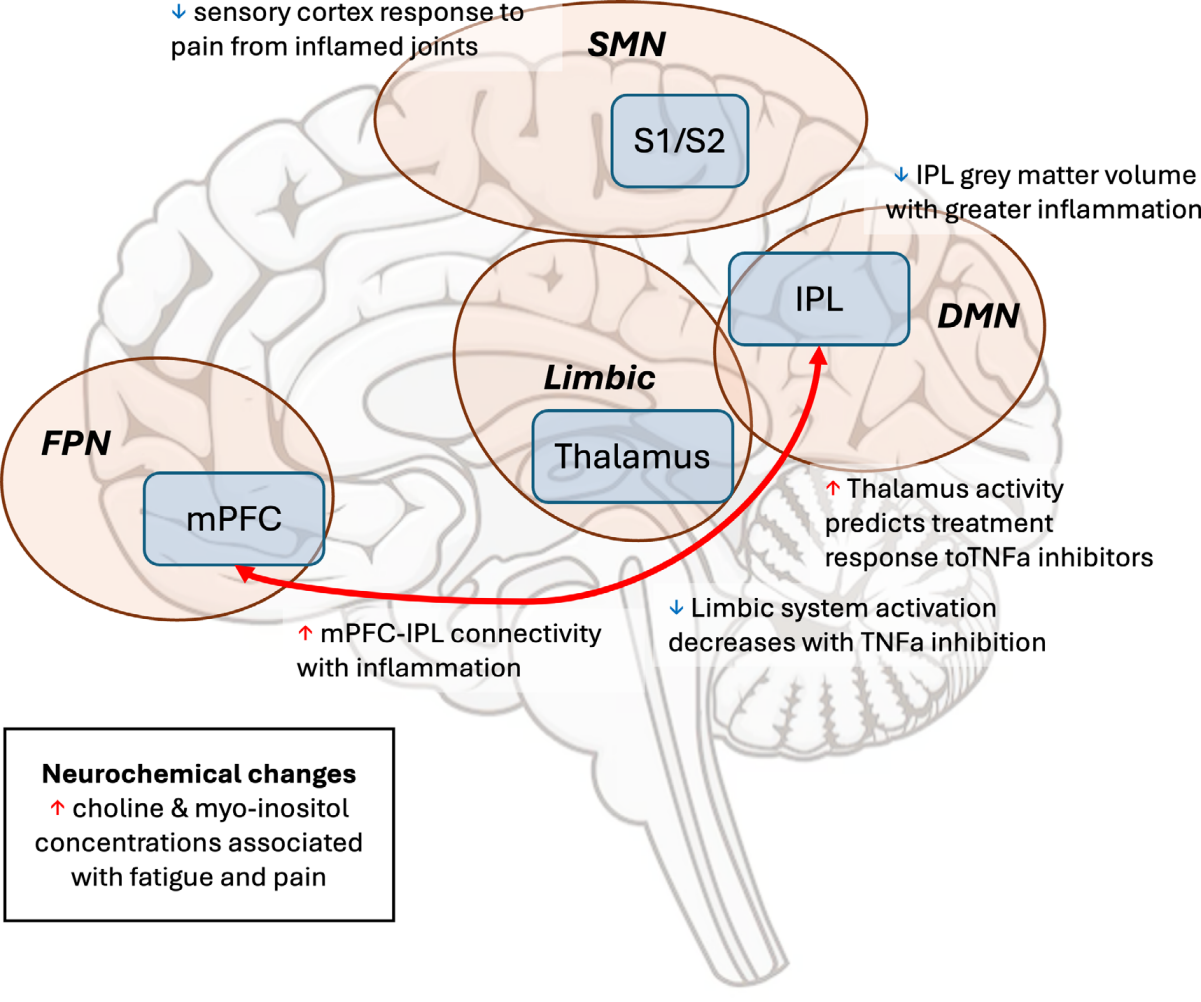
Brain changes linked to active inflammation in inflammatory arthritis. Neuroimaging studies show that active joint inflammation affects pain processing in the brain. Reduced activity in the sensory cortex (S1/S2) is seen when pain is evoked at inflamed joints. Activity in the thalamus, a key relay in pain perception, may predict response to TNF‐α inhibitors. Limbic system activity, associated with pain and fatigue, decreases after TNF blockade, even before joint swelling improves. Increased connectivity between the mPFC and IPL, a key node of the DMN, is linked to systemic inflammation. Neurochemical changes, such as higher choline and myo‐inositol, are associated with pain and fatigue, and loss of gray matter in the IPL correlates with greater inflammation. These findings demonstrate that inflammation drives both functional and structural brain changes, some of which may improve with treatment. DMN, default mode network; FPN, frontoparietal network; IPL, inferior parietal lobule; mPFC, medial prefrontal cortex; SMN, sensorimotor network; TNF‐α, tumor necrosis factor α. Color figure can be viewed in the online issue, which is available at http://onlinelibrary.wiley.com/doi/10.1002/acr.25694/abstract.

Growing evidence suggests that peripheral inflammation in RA and related conditions communicates directly with the CNS, shaping both functional connectivity and neurochemical signatures (Figure 2). The resulting brain changes help explain why patients frequently experience fatigue, mood disturbance, and pain even in the absence of overt synovitis.

### Neuroinflammation and immune‐to‐brain signaling

Peripheral inflammatory mediators, including TNF‐α, interleukin (IL)‐1, and IL‐6, can signal to the CNS via multiple pathways: humoral transport across the blood‐brain barrier, activation of afferent vagal fibers, and stimulation of circumventricular organs. These signals may promote microglial activation and subsequent release of proinflammatory cytokines within the brain; however, evidence for the presence of neural inflammation is lacking.^40^, ^41^ Chronic activation of these pathways contributes to the cluster of “sickness behaviors” (fatigue, anhedonia, and cognitive dysfunction) that typify many inflammatory rheumatic diseases.^42^

Although the concept of sickness behavior usefully encapsulates the constellation of neurovegetative and affective symptoms that accompany immune activation, accumulating evidence indicates that the physiologic and behavioral responses to inflammation evolve over time and differ between the acute and chronic phases. Work by Capuron and colleagues, among others, of patients undergoing chronic interferon‐α (IFN‐α) therapy have been particularly informative in this respect.^43^ During early treatment, patients frequently experience fatigue, psychomotor slowing, anorexia, and sleep disturbance, features that align closely with classic sickness behavior, whereas mood and cognitive symptoms such as depression, anxiety, and attentional deficits emerge more gradually with continued cytokine exposure.^44^, ^45^, ^46^, ^47^, ^48^ Neuroimaging work demonstrates that these phases map onto distinct neurobiological substrates: acute IFN‐α administration is associated with hyper‐reactivity of the hypothalamic‐pituitary‐adrenal axis, particularly in individuals who later develop major depression, whereas sustained treatment induces altered activity in fronto‐subcortical circuits.^49^ PET and fMRI studies show increased basal ganglia glucose metabolism and reduced ventral striatal responsiveness to reward, consistent with dopaminergic dysregulation underlying fatigue and anhedonia.^50^, ^51^ Concurrently, heightened activation of the ACC during cognitive tasks suggests compensatory increases in effortful control under inflammatory load.^49^ Together, these findings indicate that chronic cytokine exposure drives a progressive reorganization of central stress‐response and reward networks, distinguishing later‐phase inflammatory psychopathology from the transient, adaptive sickness response typical of acute inflammation.

### Peripheral inflammation linked to altered connectivity

fMRI studies demonstrate that systemic inflammation is associated with large‐scale alterations in resting‐state connectivity. In RA, higher ESR and C‐reactive protein (CRP) levels were linked to stronger connectivity among the mPFC, IPL, and multiple networks including the DMN, salience network, and dorsal attention network. These connectivity changes were associated with levels of fatigue, pain severity, and cognitive impairment, with the IPL appearing as a key hub in this “inflammation configuration.” Reduced gray matter volume (GMV) in the IPL was also associated with higher inflammation, indicating that systemic immune activity contributes not only to functional reorganization but also to structural changes.^36^

Beyond group‐level associations between systemic inflammation and neural activity, there is increasing recognition that the neuroimmune interface exhibits substantial interindividual variability. Even among patients with similar degrees of peripheral inflammation, central consequences may differ markedly depending on vulnerability factors within neural and immune networks. For instance, Kaplan and colleagues reported that in RA, ESR was associated with pronociceptive functional connectivity only in individuals exhibiting fibromyalgia‐like symptoms, suggesting that susceptibility to the central effects of inflammation varies among patients.^52^ Such variability may help explain the heterogeneity of pain, fatigue, and mood symptoms in rheumatic disease and supports the need for stratified, mechanism‐based approaches to therapy.

### Neurochemical correlates of systemic inflammation

MRS has provided complementary insights. Although no widespread metabolic abnormalities were identified in patients with RA compared with healthy controls, brain choline and myo‐inositol, markers of cell turnover and glial proliferation, respectively, were positively correlated with fatigue severity and with joint swelling and tenderness.^40^ These findings suggest that even in the absence of overt neuroinflammation on group comparisons, neurochemical alterations in subsets of patients may contribute to central symptoms.

### Impact of cytokine blockade on central pain processing

Neuroimaging studies have shown that anti‐TNF therapy produces rapid and measurable effects on CNS pain processing. For example, blockade of TNF‐α leads to reductions in the activation of limbic structures, the thalamus, and somatosensory cortex within 24 hours, often preceding measurable improvements in joint swelling.^38^ A recent preprint has shown similar findings, with baseline thalamic activation predicting clinical response, raising the possibility that neuroimaging could be used as a biomarker for treatment stratification.^53^ Similarly, Abe and colleagues demonstrated that aberrant functional connectivity between the ACC and insula was associated with treatment response across both RA and SpA, highlighting shared central mechanisms in inflammatory arthritis.^54^

In addition, TNF inhibition has been associated with structural brain changes in other rheumatic conditions. Wu and colleagues demonstrated a correlation between pain reduction in patients with AS and alterations in cortical thickness.^55^ Specifically, greater pain reduction was associated with cortical thickening in the left orbitofrontal cortex and thinning in the right somatosensory cortex, as measured by structural MRI. These cortical changes may relate to the emotional and sensory aspects of pain, respectively, with orbitofrontal thickening linked to emotional pain processing and somatosensory thinning associated with central sensitization.^56^, ^57^

Building on the evidence for the central effects of TNF blockade, JAK inhibitors have also demonstrated striking analgesic benefits in RA, further highlighting the potential for immune‐targeted therapies to modulate pain beyond their anti‐inflammatory effects.^58^ Clinical trials of tofacitinib, baricitinib, and upadacitinib consistently show rapid reductions in pain, in some cases greater than with TNF inhibitors.^59^ Importantly, post hoc analyses indicate that these improvements are only partly explained by reductions in swollen joints or markers of inflammation. For example, in the RA‐BEAM trial, baricitinib produced greater pain relief than adalimumab despite comparable control of synovitis, suggesting additional mechanisms influencing central pain pathways.^60^ Similarly, Dougados and colleagues reported that tofacitinib reduced residual pain, even in patients who had already achieved low disease activity or remission, supporting analgesic effects independent of overt inflammation.^61^ These clinical findings are consistent with preclinical evidence that proinflammatory cytokines such as TNF‐α, IL‐6, and IL‐17 can directly sensitize nociceptive neurons and promote both peripheral and central sensitization, thereby sustaining hyperalgesia independent of joint swelling.^18^ Although direct neuroimaging data on JAK inhibition remain lacking, these clinical observations parallel the CNS changes seen with TNF blockade and raise the possibility that JAK inhibitors also attenuate pronociceptive central signaling. By targeting multiple cytokine pathways converging on JAK/STAT signaling, these agents may influence both bottom‐up pathways driven by peripheral inflammation and top‐down mechanisms of central sensitization. Future neuroimaging studies are needed to determine whether JAK inhibitors normalize pain‐related network activity in a manner similar to TNF‐α blockade.

### Clinical implications

Together, these findings demonstrate that systemic inflammation shapes brain circuits underlying pain, fatigue, and cognition, and that modulation of these circuits may underlie part of the benefit of biologic therapy. For clinicians, this explains why patients may report rapid symptomatic improvement following cytokine blockade, sometimes even before peripheral measures of inflammation have fully resolved. They also suggest that persistent CNS changes may perpetuate symptoms despite good control of synovitis, reinforcing the need for mechanism‐based approaches to pain management in inflammatory arthritis.

## What happens in the brain when we treat the joints?

Although most pharmacologic therapies for inflammatory arthritis target immune pathways within the joints, neuroimaging studies demonstrate that their benefits can also extend to the CNS. A small but growing body of research has examined how biologic DMARDs, particularly TNF‐α inhibitors, modulate brain circuits involved in pain perception.

### Rapid central effects of TNF‐α blockade

Early fMRI work highlighted that TNF‐α blockade can rapidly normalize pain‐related brain activity, often before clinical measures of joint inflammation improve. Hess and colleagues observed a significant reduction in activity in the thalamus, insula, and ACC within 24 hours of infliximab infusion in patients with RA with active disease.^38^ Similarly, Rech and colleagues reported that responders to certolizumab pegol demonstrated pronounced reductions in the activation of the somatosensory cortex, dlPFC, and limbic structures as early as 3 days after treatment.^37^ These early CNS changes occurred well before reductions in Disease Activity Scores in 28 joints scores were evident, suggesting that the CNS is highly responsive to cytokine blockade.

### Differentiating responders and nonresponders

Neuroimaging has also provided insight into why some patients respond to TNF‐α inhibitors and others do not. Rech and colleagues showed that TNF‐α inhibitor responders exhibited greater baseline pain‐related CNS activation compared with nonresponders.^37^ After treatment, responders showed progressive declines in activation across the pain matrix, whereas nonresponders had persistently elevated activity, particularly in the thalamic and periaqueductal gray regions associated with descending modulation. Functional connectivity analyses further revealed that responders had more adaptive changes in thalamic‐insula and thalamic‐ACC connectivity, whereas nonresponders maintained maladaptive patterns.

In a similar vein, Espartal and colleagues conducted a small proof‐of‐concept study to assess the use of fMRI to evaluate pain in response to TNF‐α inhibitors in six patients with PsA.^62^ In this study, patients exhibiting a poor treatment response showed baseline activation of the sensory motor cortex. In contrast, patients with a strong treatment response did not have any baseline activation and showed activation in the sensory motor cortex, amygdala, insula, frontal, and prefrontal areas during the following week of treatment. Patients with poor treatment response showed minimal change, but larger studies are needed to verify these findings.

One of the earliest studies to demonstrate the utility of fMRI in inflammatory arthritis was a single‐patient pharmacologic‐MRI study investigating the modulation of brain activity of PsA pain by a single dose of a COX‐2 inhibitor.^63^ In this study, the patient was a 53‐year‐old man with active PsA where it had been noted that treatment with a COX‐2 inhibitor had resulted in profound pain and rapid relief without resolution of the associated joint swelling. Brain imaging showed that activity in the anterior and posterior insula correlated with pain intensity, showing reduced activity following COX‐2 inhibitor administration. This study, albeit in a single patient, underscores the potential of fMRI as a tool for identifying central mechanisms of pain and its value in advancing mechanism‐based treatment stratification.^64^

### Connectivity predictors of treatment response

More recent studies suggest that resting‐state connectivity patterns may predict clinical outcomes. Abe and colleagues^54^ demonstrated that altered insula‐ACC connectivity at baseline was associated with subsequent treatment response in both RA and SpA, highlighting the role of salience network dysfunction across inflammatory joint conditions. In a prospective cohort, Wartolowska and colleagues^53^ reported that higher baseline thalamic activation predicted greater improvement in pain following TNF blockade, further supporting the potential utility of neuroimaging biomarkers in stratifying patients for therapy.

### Clinical implications

These findings provide a neurobiologic explanation for the frequently observed phenomenon in clinical practice that some patients report rapid pain relief shortly after initiation of TNF inhibitors, even in the absence of objective improvements in joint swelling or CRP. They also underscore that persistent pain after effective immunosuppression may reflect enduring central mechanisms rather than refractory synovitis. In the future, neuroimaging markers may help to personalize treatment decisions by identifying patients most likely to benefit from cytokine blockade and guiding the integration of adjunctive therapies targeting nociplastic pain pathways.

## Beyond pain: central mechanisms behind fatigue and mood disturbance

Although pain is the most prominent and burdensome symptom in inflammatory arthritis, patients frequently report fatigue, low mood, and reduced resilience as equally disabling.

### Depression and altered affective circuits

Neuroimaging work underscores the link between depressive symptoms and altered pain processing in inflammatory arthritis. Schweinhardt and colleagues found that in RA, activation of the mPFC during evoked joint pain correlated with greater depressive symptom scores, suggesting that affective state directly influences cerebral pain processing.^65^ Connectivity among the mPFC, posterior cingulate cortex, and other limbic structures was strengthened in patients with higher depressive symptoms, providing a neural correlate for the clinical observation that depression amplifies pain experience. Kaplan and colleagues similarly emphasized the role of limbic hyperactivation in the affective dimension of pain, reinforcing the concept that depression and pain share overlapping neurobiologic substrates.^52^

Additionally, rs‐fMRI studies can provide valuable insights into the neurobiologic mechanisms underlying the relationship between brain activity and pain and depression. Hua and colleagues investigated regional homogeneity (ReHo), a measure of functional connectivity alterations in patients with AS.^66^ Compared with healthy controls, their study revealed higher ReHo in the left precuneus and right middle frontal gyrus and reduced ReHo in the left superior temporal gyrus and right paracentral lobule. A negative correlation was observed between ReHo in the right paracentral lobule and disease duration and back pain scores. Conversely, a positive correlation was observed between ReHo in the left precuneus and depression and back pain scores.

### Fatigue and network reorganization

Fatigue is among the most disabling symptoms reported by patients with inflammatory arthritis and often persists despite good inflammatory control. Schrepf and colleagues demonstrated that systemic markers of inflammation (ESR and CRP) were associated with altered connectivity among the mPFC, IPL, and large‐scale networks such as the DMN and dorsal attention network.^36^ These connectivity changes correlated not only with pain but also with fatigue and subjective cognitive dysfunction, suggesting a shared neurobiologic basis for these symptoms. In addition, Liu and colleagues reported similar findings.^67^ In a study that investigated functional and structural alterations in patients with AS, they observed that altered functional connectivity between DMN and salience network nodes correlated with fatigue. For within DMN nodes this was correlated not only with pain but also with fatigue. Furthermore, putamen GMV was associated with pain and fatigue. Hua and colleagues also observed an increase of GMV in this subcortical brain region in patients with AS with moderate back pain; this increase correlated with disease duration.^68^

### Sex differences

The experience of pain in inflammatory arthritis is known to differ between sexes,^69^, ^70^, ^71^ but the underlying mechanistic drivers are not yet fully understood. Neuroimaging can further shed light on the distinct pain mechanisms between sexes, which in turn may help to uncover tailored treatment strategies. Fauchon and colleagues used rs‐fMRI data to conduct modular analysis using graph theory and found distinct brain modular structures in healthy male and female patients.^72^ Data from patients with AS with chronic pain revealed altered brain organization, particularly in female patients, who exhibited DMN modularity, increased salience/attention network activity, and decreased frontal network connectivity. In contrast, male patients exhibited increased intermodular connectivity, particularly between the DMN and prefrontal areas. Similarly, Rogachov and colleagues investigated the relationship between chronic pain in patients with AS and low‐frequency oscillations (LFOs) in the BOLD signal of rs‐fMRI.^73^ LFOs, which are measured at rest, reflect slow, rhythmic fluctuations in the BOLD signal, providing insights into brain network function.^74^ Compared with healthy controls, patients with AS exhibited increased LFO amplitudes and distinct LFO patterns within pain‐related brain networks. Analysis of sex differences revealed that female patients (except in the posterior cingulate/precuneus) exhibited the slowest brain frequencies in both the healthy control and AS groups. Within the AS group, male and female patients displayed different LFO patterns despite reporting similar pain levels. Overall, these findings suggest that, even if the burden of pain is similar between sexes, targeting the different underlying brain‐based mechanisms may improve overall treatment responses.

### Resilience and adaptive central responses

Emerging evidence highlights that not all patients respond to systemic inflammation in the same way. Some individuals appear to maintain functional resilience, with preserved or adaptive connectivity in frontoparietal and executive networks despite ongoing inflammation. Abe and colleagues^54^ identified that connectivity patterns between the ACC and insula not only predicted treatment response but may also reflect an individual's capacity for central compensation in the face of persistent inflammatory signaling. These findings align with broader literature on cognitive resilience, suggesting that variability in network plasticity and descending pain modulatory capacity may partially explain why some patients adapt better to chronic disease burden than others. Interestingly, psychological resilience has also been linked with pain‐related functional connectivity.^75^ Hemington and colleagues found that resilience scores of healthy control patients showed a negative correlation with DMN‐salience network internetwork connectivity. In contrast, patients with AS had a positive correlation between pain scores and interconnectivity between these networks.

### Clinical implications

Together, these studies extend the neuroimaging evidence in inflammatory arthritis beyond pain to encompass fatigue, depression, and resilience. For clinicians, they reinforce that persistent fatigue or mood disturbance should not be dismissed as nonspecific complaints but rather as potential markers of central dysregulation linked to inflammatory signaling. This recognition may encourage integration of mental health support, fatigue‐management strategies, and centrally acting pharmacologic or nonpharmacologic interventions into standard care alongside immunosuppressive therapy.

## Integrating the pathways: a mechanism‐based model for pain management in inflammatory arthritis

**Figure 3:**
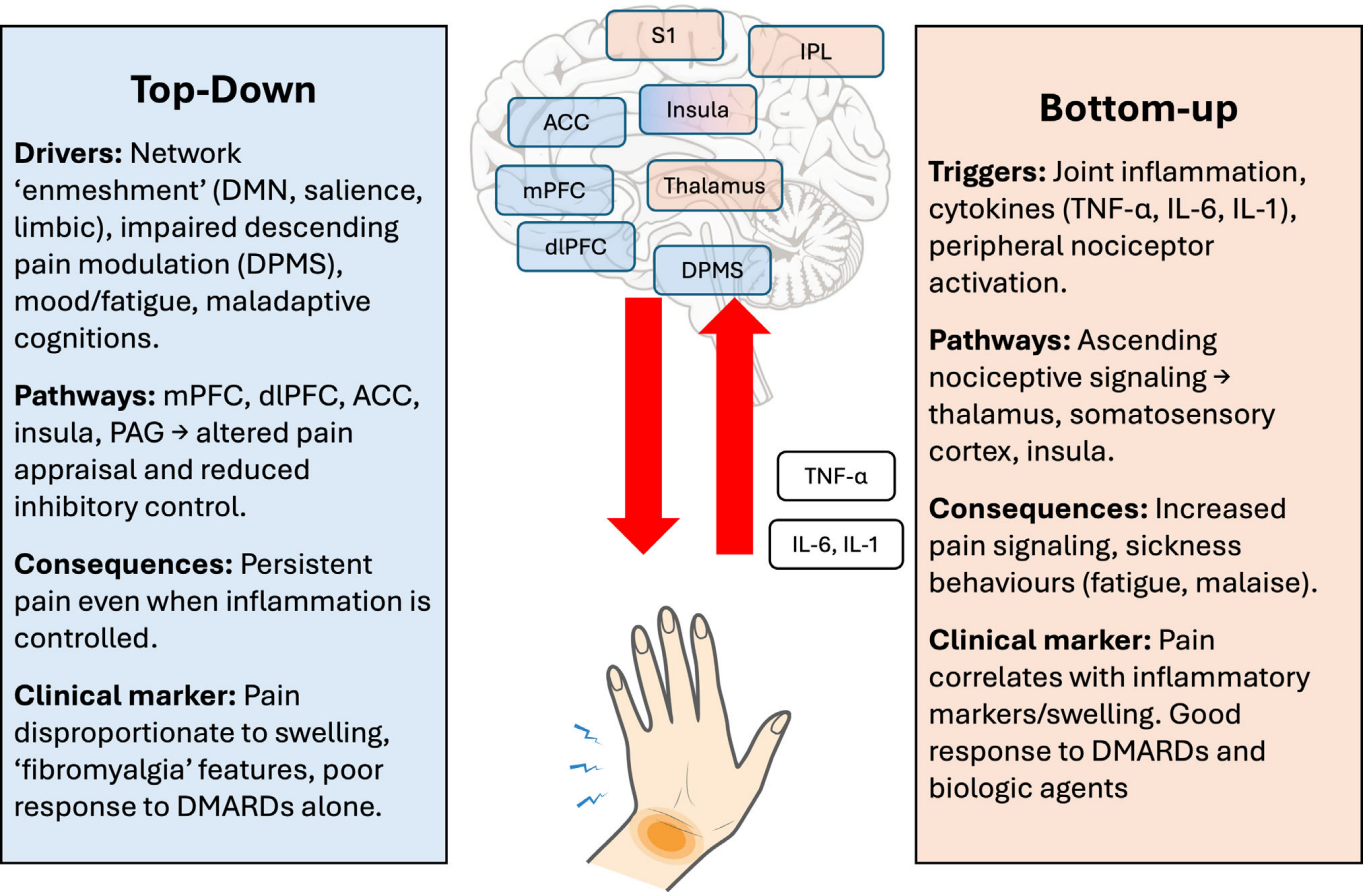
Top‐down and bottom‐up mechanisms of persistent pain in inflammatory arthritis. Neuroimaging evidence highlights two complementary pathways that contribute to pain beyond joint inflammation. Bottom‐up mechanisms involve peripheral drivers such as joint inflammation and cytokines (TNF‐α, IL‐6, and IL‐1) that activate ascending nociceptive pathways to the thalamus, somatosensory cortex, and insula, producing increased pain signaling and sickness behaviors. This phenotype is characterized by pain that correlates with markers of inflammation and typically improves with DMARDs or biologics. In contrast, top‐down mechanisms reflect central sensitization and maladaptive modulation of pain, driven by the enmeshment of the default mode, salience, and limbic networks, impaired DPMS activity, and mood/fatigue‐related processes. These changes, involving the mPFC, ACC, insula, and PAG, sustain pain despite controlled inflammation. Clinically, this presents as pain disproportionate to swelling, fibromyalgia‐like features, and poor response to DMARDs alone. Together, these pathways emphasize the dual central and peripheral origins of persistent pain in inflammatory arthritis. ACC, anterior cingulate cortex; dlPFC, dorsolateral prefrontal cortex; DMARD, disease‐modifying antirheumatic drug; DMN, default mode network; DPMS, descending pain modulatory system; IL, interleukin; IPL, inferior parietal lobule; mPFC, medial prefrontal cortex; PAG, periaqueductal gray; TNF‐α, tumor necrosis factor α. Color figure can be viewed in the online issue, which is available at http://onlinelibrary.wiley.com/doi/10.1002/acr.25694/abstract.

The accumulating neuroimaging evidence supports a reframing of pain in inflammatory arthritis as a product of both bottom‐up inflammatory signaling and top‐down CNS modulation (Figure 3). Although DMARDs remain the cornerstone of treatment for synovitis, many patients continue to experience pain, fatigue, and mood disturbance after inflammatory control is achieved. This disconnect highlights the need for a mechanism‐based model of pain management that integrates immune modulation with strategies targeting central pain pathways.

### A top‐down and bottom‐up framework

In this model, peripheral inflammation acts as a bottom‐up driver of pain, activating ascending nociceptive pathways via cytokine‐mediated effects on the thalamus, insula, and somatosensory cortices.^36^, ^38^ At the same time, maladaptive top‐down processes, including hyperconnectivity of the DMN, salience network, and limbic network and impaired DPMS activity, sustain nociplastic pain even when inflammation is controlled.^23^, ^37^ Importantly, these processes are not mutually exclusive but frequently coexist, producing the complex pain phenotypes observed in RA, PsA, and axSpA.

### Opportunities for CNS‐targeted interventions

Recognizing the central contribution to pain in inflammatory arthritis opens the door for adjunctive therapies targeting CNS pathways.

- Pharmacologic interventions: centrally acting medications such as serotonin‐noradrenaline reuptake inhibitors (eg, duloxetine) have demonstrated efficacy in fibromyalgia and may benefit patients with inflammatory arthritis with nociplastic pain features.^52^ Other agents, including pregabalin and low‐dose tricyclic antidepressants, may also be considered in select cases.
- Psychological therapies: cognitive behavioral therapy and acceptance and commitment therapy have been shown to improve coping, reduce catastrophizing, and enhance resilience, with fMRI studies suggesting they modulate the frontoparietal and limbic circuits implicated in nociplastic pain.^65^
- Lifestyle and nonpharmacologic approaches: exercise, sleep optimization, and stress‐reduction strategies (eg, mindfulness‐based interventions) have demonstrated benefits for both pain and fatigue. Neuroimaging evidence suggests these interventions enhance functional connectivity within executive and descending inhibitory networks, potentially restoring more adaptive top‐down regulation.

### Toward mechanism‐based personalized care

Future clinical models should incorporate systematic screening for nociplastic pain features, fatigue, and mood disturbance alongside traditional disease activity assessments. Neuroimaging biomarkers, including baseline thalamic and insula‐ACC connectivity patterns, may eventually guide treatment stratification by identifying patients most likely to benefit from biologic therapies or adjunctive CNS‐targeted interventions.^38^, ^39^, ^53^, ^54^ Such an integrated, mechanism‐based approach shifts the paradigm from treating to target to treating to recovery, with the ultimate goal of reducing pain, improving function, and enhancing quality of life.

## Putting the brain on the rheumatology roadmap

The recognition that CNS mechanisms play a fundamental role in pain, fatigue, and mood disturbance in inflammatory arthritis represents a paradigm shift for rheumatology. Neuroimaging has moved this field forward by providing objective, in vivo markers of central pain processing and demonstrating that brain changes both respond to, and sometimes predict, treatment effects. To realize the clinical potential of these insights, several key directions are now needed.

### Larger, longitudinal cohort studies

Most existing neuroimaging studies in inflammatory arthritis have been small, single‐center investigations. Larger, longitudinal cohorts will be essential to validate early findings and determine the natural history of CNS changes in relation to disease activity, treatment, and patient‐reported outcomes. Such studies could clarify whether specific neuroimaging signatures are stable traits, modifiable states, or both and whether they can predict long‐term functional outcomes.^23^, ^36^

### Neuroimaging as a stratification tool for clinical trials

There is growing interest in using neuroimaging biomarkers to stratify patients in randomized controlled trials. Baseline thalamic activation and insula‐ACC connectivity, for example, have been linked to differential responses to TNF inhibitors.^37^, ^54^ Incorporating such markers into trial design could help identify the patients most likely to benefit from specific biologics or adjunctive centrally acting therapies, increasing both the efficiency and success of trials.

### Back‐translation of mechanistic insights to clinical measures

To ensure that mechanistic insights reach the bedside, there is a need to translate neuroimaging findings into accessible clinical tools. This could include the refinement of composite indices that integrate measures of nociplastic pain features, fatigue, and mood disturbance, informed by brain‐based evidence. Routine use of validated patient‐reported outcome measures, alongside clinical and laboratory indices, may allow rheumatologists to better identify patients with centrally mediated symptoms, even when advanced imaging is not available.^52^, ^53^

### The future of mechanism‐based rheumatology

Ultimately, the integration of brain‐focused research into rheumatology practice promises a more comprehensive, mechanism‐based approach to patient care. By recognizing the CNS as both a target and a mediator of symptom burden, the field can move beyond treating inflammation alone to improving holistic outcomes. Neuroimaging stands to play a pivotal role in this transformation, guiding patient stratification, informing personalized therapy, and closing the gap between inflammatory control and meaningful recovery.

## AUTHOR CONTRIBUTIONS

All authors contributed to at least one of the following manuscript preparation roles: conceptualization AND/OR methodology, software, investigation, formal analysis, data curation, visualization, and validation AND drafting or reviewing/editing the final draft. As corresponding author, Dr Kelleher confirms that all authors have provided the final approval of the version to be published and takes responsibility for the affirmations regarding article submission (eg, not under consideration by another journal), the integrity of the data presented, and the statements regarding compliance with institutional review board/Declaration of Helsinki requirements.

## Supporting information


**Disclosure form**.
